# Effects of Aging and Caloric Restriction on Fiber Type Composition, Mitochondrial Morphology and Dynamics in Rat Oxidative and Glycolytic Muscles

**DOI:** 10.3389/fphys.2019.00420

**Published:** 2019-04-24

**Authors:** Julie Faitg, Jean-Philippe Leduc-Gaudet, Olivier Reynaud, Guylaine Ferland, Pierrette Gaudreau, Gilles Gouspillou

**Affiliations:** ^1^ Département de Biologie, Faculté des Sciences, UQAM, Montreal, QC, Canada; ^2^ Groupe de recherche en Activité Physique Adaptée, Montreal, QC, Canada; ^3^ Meakins-Christie Laboratories, Department of Medicine and Division of Experimental Medicine, McGill University, Montreal, QC, Canada; ^4^ Département des sciences de l’activité physique, Faculté des Sciences, UQAM, Montreal, QC, Canada; ^5^ Institut de cardiologie de Montréal Research Center, Montreal, QC, Canada; ^6^ Department of Nutrition, University of Montreal, Montreal, QC, Canada; ^7^ Laboratory of Neuroendocrinology of Aging, Centre Hospitalier de l’Université de Montréal Research Center (CRCHUM), Montreal, QC, Canada; ^8^ Department of Medicine,University of Montreal, Montreal, QC, Canada; ^9^ Centre de Recherche de l’Institut Universitaire de Gériatrie de Montréal, Montreal, QC, Canada

**Keywords:** aging, nutrition, sarcopenia, atrophy, skeletal muscle, electron microscopy

## Abstract

Aging is associated with a progressive decline in muscle mass and strength, a process known as sarcopenia. Evidence indicates that mitochondrial dysfunction plays a causal role in sarcopenia and suggests that alterations in mitochondrial dynamics/morphology may represent an underlying mechanism. Caloric restriction (CR) is among the most efficient nonpharmacological interventions to attenuate sarcopenia in rodents and is thought to exert its beneficial effects by improving mitochondrial function. However, CR effects on mitochondrial morphology and dynamics, especially in aging muscle, remain unknown. To address this issue, we investigated mitochondrial morphology and dynamics in the oxidative soleus (SOL) and glycolytic white gastrocnemius (WG) muscles of adult (9-month-old) *ad libitum*-fed (AL; A-AL), old (22-month-old) AL-fed (O-AL), and old CR (O-CR) rats. We show that CR attenuates the aging-related decline in the muscle-to-body-weight ratio, a sarcopenic index. CR also prevented the effects of aging on muscle fiber type composition in both muscles. With aging, the SOL displayed fragmented SubSarcolemmal (SS) and InterMyoFibrillar (IMF) mitochondria, an effect attenuated by CR. Aged WG displayed enlarged SS and more complex/branched IMF mitochondria. CR had marginal anti-aging effects on WG mitochondrial morphology. In the SOL, DRP1 (pro-fission protein) content was higher in O-AL vs YA-AL, and Mfn2 (pro-fusion) content was higher in O-CR vs A-AL. In the gastrocnemius, Mfn2, Drp1, and Fis1 (pro-fission) contents were higher in O-AL vs A-AL. CR reduced this aging-related increase in Mfn2 and Fis1 content. Overall, these results reveal for the first time that aging differentially impacts mitochondrial morphology and dynamics in different muscle fiber types, by increasing fission/fragmentation in oxidative fibers while enhancing mitochondrial size and branching in glycolytic fibers. Our results also indicate that although CR partially attenuates aging-related changes in mitochondrial dynamics in glycolytic fibers, its anti-aging effect on mitochondrial morphology is restricted to oxidative fibers.

## Introduction

Skeletal muscle aging is associated with a progressive decline in muscle mass and function, a biological process known as sarcopenia (Rosenberg, 1997). Several lines of evidence indicate that there is a sustained increase in mitochondrial dysfunction with aging in rodents and humans, which contributes to the progressive decline of muscle mass and function (Dirks and Leeuwenburgh, 2002, 2005; Drew and Leeuwenburgh, 2003; Short et al., 2005; Marzetti et al., 2008; Gouspillou et al., 2010, 2014a). More specifically, aged skeletal muscles display impaired mitochondrial bioenergetics (Drew and Leeuwenburgh, 2003; Short et al., 2005; Gouspillou et al., 2010) and an increase in mitochondrially mediated apoptosis (Dirks and Leeuwenburgh, 2002; Marzetti et al., 2008; Gouspillou et al., 2014a), which primarily contributes to the aging-related decline in muscle mass and function. The implication of mitochondrial dysfunction in the sarcopenic process is further supported by the fact that overexpression of the mitochondrial-targeted catalase (an antioxidant enzyme) attenuates the loss of muscle mass occurring with aging (Umanskaya et al., 2014).

Long-term caloric restriction (CR) is among the most efficient interventions to offset sarcopenia in rodents, and CR is thought to exert its effects mostly through by improving mitochondria (see (Gouspillou and Hepple, 2013) for review). Indeed, CR has been shown to decrease mitochondrial reactive oxygen species (ROS) production (Asami et al., 2008; Lanza et al., 2012), increase mitochondrial coupling efficiency (Lanza et al., 2012), and increase mitochondrial respiration per unit of mitochondria in aged muscles (Hepple et al., 2005, 2006). CR has also been shown to reduce markers of apoptosis in aged rat skeletal muscle (Wohlgemuth et al., 2010). However, many of the effects of CR, specifically on mitochondrial biology, in aging muscles, remain to be studied. Thus, the impact of CR on mitochondrial dynamics and morphology has never been investigated in adult and old muscles. This is of particular importance since it is now established that mitochondrial morphology and function are interrelated, with changes in mitochondrial morphology affecting mitochondrial function (Chen et al., 2005; Jahani-Asl et al., 2007; Yu et al., 2008; Ong et al., 2010; Gomes et al., 2011) and *vice versa* (Benard et al., 2007).

In most cell types, and particularly in skeletal muscle (Ogata and Yamasaki, 1997), mitochondria display a complex and dynamic architecture. Indeed, mitochondria form a dynamic network, with the ability to undergo fusion and fission events, processes collectively known as mitochondrial dynamics (Chan, 2006), which are regulated by Mitofusins 1 & 2 (Mfn 1 & 2) and OPtic Atrophy 1 (OPA-1) and Dynamin-Related Protein 1 (DRP1) and mitochondrial-FISsion 1 protein (FIS1) proteins, respectively (Chan, 2006). It was for instance shown in skeletal muscle of young animals that Mfn-1 and -2 deletion leads to abnormal mitochondrial morphology, severe mitochondrial dysfunction, and a severe deficit in muscle growth (Chen et al., 2010). Mitochondrial morphology and dynamic therefore play important roles in mitochondrial and skeletal muscle physiology. However, to date, the effects of aging on mitochondrial morphology and dynamics in skeletal muscle remain unclear, and those of CR are currently unknown.

In the present study, we investigated the morphology of sub-sarcolemmal (SS) and Intermyofibrillar (IMF) mitochondria in the glycolytic white gastrocnemius and the oxidative soleus muscles of adult and old rats using a 2-dimensional transmission electron microscopy approach (Picard et al., 2013a,b), in order to get a better understanding of the impact of aging and caloric restriction on mitochondrial morphology in skeletal muscles. As we previously observed (Leduc-Gaudet et al., 2015) that the white gastrocnemius of aged mice display larger and less circular SS mitochondria and longer and more branched IMF mitochondria, we hypothesized that (1) aging in rats would result in an increased mitochondrial size and morphological complexity associated with elevated expression of fusion proteins and/or decreased expression of fission proteins in both glycolytic and oxidative skeletal muscle and (2) CR would attenuate the effects of aging on mitochondrial morphology and dynamics.

## Materials and Methods

### Animals and Tissue Collection

All animal procedures were approved by the *Comité Institutionnel de Protection des Animaux de l’UQAM* (#CIPA875) and the *Centre de recherche de l’hôpital du Sacré-Coeur de Montréal* (#FRSQ.01) in compliance with the guidelines of the Canadian Council on Animal Care. All experiments were performed on male Sprague–Dawley rats, obtained either from Charles River (St-Constant, Québec, Canada) or the Quebec Network for Research on Aging (QNRA). Nine-month-old adults *ad libitum-fed* (A-AL, n = 9; Charles River Canada, St-Constant, QC), 22-month-old *ad libitum*-fed (O-AL, n = 4, which represented all available O-AL rats through the QRNA) and 22-month-old rats submitted to CR for 13 months (O-CR, n = 11; QNRA) rats were studied. CR was initiated at 8 months of age, starting with a 20% restriction for 2 weeks, followed by a 40% CR for 13 months. Rats from both O-AL and O-CR groups were fed an AIN-93 based diet (Prot 22%, Carb 63%, Fat 15%; Harland, Teklad, Madison, WI). Animals from the O-AL group received standard diet (TD.130770) throughout their lives, whereas rats from the O-CR group were administered this diet until the age of 8 months. At that stage, which corresponds to the start of the CR regimen, O-CR animals received a diet enriched in vitamins and minerals (TD.130771) to ensure that both groups received comparable amounts of micronutrients. A-AL rats were fed a comparable control diet (Charles River Rodent Diet # 5075, St-Constant, QC, Canada; composition: Prot 21%, Carb 66%, Fat 13%).

The animals were euthanized by rapid decapitation. The soleus (SOL) and gastrocnemius (GAS) muscles from left hind paws were collected. Both muscles were cut in half. A piece of the white portion of the GAS (WG) was isolated from the rest of the first half of the GAS. This portion of the WG and the first half of the SOL were sliced into small pieces (>1 mm in thickness) and prepared for TEM analysis. The rest of the SOL and the second half of the GAS were snap frozen in liquid nitrogen for Western blotting and stored at −80°C until use. The GAS and SOL from the right leg were removed. For each of these muscles, a slice of the entire mid-belly was mounted on a cork in optimal cutting temperature compound and frozen in liquid isopentane cooled in liquid nitrogen. Histology samples were stored at −80°C until use. The tibialis anterior (TA), extensor digitorum longus (EDL), and plantaris (PL) muscles from both legs were finally isolated and weighed to obtain a comprehensive assessment of the impact of aging and CR on muscle mass.

### Transmission Electron Microscopy

Muscle samples were fixed in 2% glutaraldehyde solution 0.1 M cacodylate buffer (pH 7.4). These samples were later postfixed in 1% osmium tetroxyde during 1 h, dehydrated within an increasing concentration of acetone. To verify the orientation of the muscle tissue, 1 μm thick sections were stained with toluidine blue prior to ultrathin sectioning. Ultrathin sections were cut in a longitudinal or transverse orientation on an Ultracut ultramicrotome (Leica). These sections were then stretched to remove compressions and mounted on copper grids before being stained with 2% aqueous uranyl acetate and lead citrate (Leica). Sections were then imaged using a Philips CM 100 electron microscope. Digital micrographs were captured using an AMT XR80 CCD digital camera at ×7900 magnification.

Individual SS and IMF mitochondria from 4 A-AL, 4 O-AL, and 4 O-CR rats were manually traced in longitudinal and transverse orientations using ImageJ software (NIH) (https://imagej.nih.gov/ij/) and to quantify the following morphological and shape descriptors: area (μm^2^), perimeter (μm), circularity 4π × (area/perimeter^2^), aspect ratio ((major axis)/(minor axis)): the aspect ratio being a measure of the length to width ratio, form factor ((perimeter) / (4π × surface)): a measure sensitive to the complexity and the branched appearance of the mitochondria, Feret’s diameter (the longest distance (μm) between two points of the mitochondria studied) and minimum Feret diameter (corresponds to the smallest diameter of the mitochondria) (Picard et al., 2012). It is important to highlight here that n = 3–4 is a sample size commonly used in TEM-based studies investigating mitochondrial morphology and ultrastructure (Bonnard et al., 2008; Sato et al., 2013; Arruda et al., 2014; Leduc-Gaudet et al., 2015; Saleem et al., 2015; Demeter-Haludka et al., 2018; Loro et al., 2018). Details on the number of SS and IMF mitochondria that were traced in both orientations in soleus and white gastrocnemius are available in Table 1.

**Table 1:** Effects of aging and caloric restriction on morphological parameters and shape descriptors of subsarcolemmal and intermyofibrillar mitochondria in the soleus.

Soleus						
	Longitudinal orientation			Transverse orientation		
	A-AL	O-AL	O-RC	A-AL	O-AL	O-RC
**SS**
N	1,612	4,746	1,359	1,993	1,880	1,143
Area (um^2^)	0.25 ± 0.006^a^^,^^b^	0.19 ± 0.002^a^^,^^c^	0.23 ± 0.006^b^^,^^c^	0.22 ± 0.004	0.22 ± 0.005	0.24 ± 0.007
Perimeter (μm)	1.81 ± 0.022^a^	1.64 ± 0.011^a^^,^^c^	1.76 ± 0.023^c^	1.73 ± 0.017	1.72 ± 0.018	1.82 ± 0.026
Circularity	0.84 ± 0.003	0.84 ± 0.002	0.84 ± 0.004	0.84 ± 0.003^a^	0.85 ± 0.003^a^^,^^c^	0.83 ± 0.005^c^
Aspect ratio	1.71 ± 0.017	1.69 ± 0.010	1.70 ± 0.018	1.75 ± 0.017	1.68 ± 0.017	1.76 ± 0.025
Form factor	1.24 ± 0.007	1.25 ± 0.005	1.25 ± 0.008	1.25 ± 0.007	1.22 ± 0.008^c^	1.27 ± 0.011^c^
Minimum Feret (μm)	0.42 ± 0.005^a^	0.38 ± 0.002^a^^,^^c^	0.41 ± 0.005^c^	0.40 ± 0.004	0.41 ± 0.004	0.42 ± 0.005
**IMF**
N	3,955	8,487	6,101	3,189	7,138	3,962
Area (um^2^)	0.10 ± 0.002^a^	0.11 ± 0.001^a^^,^^c^	0.1 ± 0.001^c^	0.14 ± 0.002^a^^,^^b^	0.09 ± 0.001^a^^,^^c^	0.15 ± 0.002^b^^,^^c^
Perimeter (μm)	1.21 ± 0.009^a^	1.26 ± 0.008^a^^,^^c^	1.18 ± 0.008^c^	1.78 ± 0.021^a^^,^^b^	1.24 ± 0.009^a^^,^^c^	1.67 ± 0.019^b^^,^^c^
Circularity	0.81 ± 0.002^b^	0.81 ± 0.002^c^	0.83 ± 0.002^b^^,^^c^	0.64 ± 0.005^a^^,^^b^	0.77 ± 0.002^a^^,^^c^	0.72 ± 0.004^b^^,^^c^
Aspect ratio	1.84 ± 0.013^a^^,^^b^	1.89 ± 0.011^a^^,^^c^	1.76 ± 0.010^b^^,^^c^	3.02 ± 0.035^a^^,^^b^	2.17 ± 0.016^a^^,^^c^	2.50 ± 0.028^b^^,^^c^
Form factor	1.28 ± 0.005^a^^,^^b^	1.31 ± 0.004^a^^,^^c^	1.26 ± 0.004^b^^,^^c^	1.99 ± 0.021^a^^,^^b^	1.45 ± 0.008^a^^,^^c^	1.68 ± 0.017^b^^,^^c^
Minimum Feret (μm)	0.26 ± 0.002	0.27 ± 0.001	0.26 ± 0.001	0.28 ± 0.002^a^^,^^b^	0.25 ± 0.001^a^^,^^c^	0.30 ± 0.002^b^^,^^c^

Data were obtained from adult ad libitum-fed (A-AL, N = 4), old ad libitum-fed (O-AL, N = 4) and old CR rats (O-CR, N = 4). Data are presented as mean ± SEM.

a *p* < 0.05 A-AL vs O-AL;

b *p* < 0.05 A-AL vs O-CR;

c *p* < 0.05 O-AL vs O-CR.

### Muscle Sections for Histology Analysis

Eight-micron thick serial cross-sections were cut in a cryostat at −18°C and mounted on lysine coated slides (Superfrost) to determine fiber type and mitochondrial content as previously described (Gouspillou et al., 2014a,b).

### Fiber Typing in Muscle Sections

Muscle cross-sections were immunolabeled at the same time for the different myosin heavy chains (MHC) as previously described (Leduc-Gaudet et al., 2015). Briefly, sections were rehydrated with PBS (pH 7.2), blocked using goat serum (10% PBS), and incubated for 1 h at room temperature with the following primary antibody cocktail: a mouse IgG2b monoclonal anti-MHC type I (BA-F8, 1:25), mouse IgG1 monoclonal anti-MHC type IIa (SC-71, 1:200), mouse IgM monoclonal anti-MHC type IIb (BF-F3, 1:200), and a rabbit IgG polyclonal anti-laminin (Sigma L9393, 1: 750). Muscle cross sections were then washed three times in PBS before being incubated for 1 h with the following secondary antibody cocktail: Alexa Fluor 350 IgG2b (y2b), goat anti-mouse antibody (Invitrogen, A-21140, 1: 500), Alexa Fluor 594 IgG1 (y1) goat anti-mouse (Invitrogen, A-21125, 1:100), Alexa Fluor 488 IgM goat anti-mouse (Invitrogen, A-21042, 1:500), and Alexa Fluor 488 IgG goat anti-rabbit (A-11008, 1:500). These sections were then washed three times in PBS and cover slipped using Prolong Gold (P36930; Invitrogen) as mounting medium. All primary antibodies targeting MHCs were purchased from the Developmental Studies Hybridoma Bank (DSHB, University of Iowa, IA). Slides were imaged using a Zeiss Axio Imager 2 fluorescence microscope (Zeiss, Oberkochen, Germany).

### *In situ* Determination of the Succinate Dehydrogenase Activity

Sections were stained for succinate dehydrogenase (SDH, complex II of the respiratory chain) activity. Muscle cross-sections were first allowed to reach room temperature. Sections were then incubated in a solution containing nitroblue tetrazolium (1.5 mM), sodium succinate (130 mM), phenazine methosulphate (0.2 mM), and sodium azide (0.1 mM) for 10 min at room temperature. Cross sections were then washed 3 times (3 × 5min) in distilled water and cover-slipped using an aqueous mounting medium (Vector Labs, VectaMount AQ Medium, H-5501). All samples for each species were processed at the same time and using the same incubation solution, ensuring that all samples underwent the exact same experimental conditions.

### Immunoblotting

The protein levels of Mfn2, Drp1 and Fis1 were determined in muscle homogenates prepared from SOL and GAS muscles. Approximately 10 mg of each muscle were homogenized in 10 volumes of an extraction buffer composed of Tris base 50 mM, NaCl 150 mM, Triton X-100 1%, Sodium deoxycholate 0.5%, SDS 0.1%, and 10 μl/ml of a protease inhibitor cocktail (Sigma P8340). The homogenate was centrifuged at 15,000 *g* for 15 min at 4°C. Protein content in the supernatant was determined using the Bradford method.

Aliquots of supernatant were mixed with Laemmli buffer and subsequently boiled at 95°C for 5 min. Thirty micrograms of proteins for each sample were loaded onto gradient (4–15%) and stain-free gels (Mini PROTEAN® TGX Stain-Free TM Gels, Biorad), electrophoresed by SDS-PAGE, and then transferred to polyvinylidene fluoride membranes (PVDF, Biorad). A stain-free blot image was taken using the ChemiDoc™ Touch Imaging System for total protein measurement in each sample lane. Membranes were blocked in 5% nonfat milk in Tris-Buffered Saline containing 0.1% Tween 20 (TBS-T) for 1 h at room temperature and then probed for 1 h using the following antibodies: anti-Mfn2 (Abcam, ab50843, 1:1000), anti-DRP1 (Abcam, ab56788, 1:1000), and anti-Fis1 (Life science, ALX210–907, 1:1000). All antibodies were diluted in blocking buffer. Membranes were then washed 6 times for 5 min each in TBS-T and subsequently incubated with HRP-conjugated secondary antibodies (Abcam Ab6728 or Ab6721, 1:5000) diluted in blocking buffer 1 h at room temperature. Signals were detected using enhanced chemiluminescence substrate (Biorad, Clarity ECL substrate, 170–5,060) using the ChemiDoc™ Touch Imaging System. All images were analyzed using the ImageLab software (Biorad). For each sample, the ECL signal for the protein of interest was normalized to the intensity of the stain-free blot image of the corresponding sample (i.e., the intensity of the stain-free blot image was used as loading control) (Rivero-Gutierrez et al., 2014).

### Statistical Analyses

Differences in animal body weight, mitochondrial density by TEM, average fiber size, and average values of shape descriptions used to assess mitochondrial morphology were analyzed using an Ordinary one–way ANOVA followed by post-hoc tests using the two-stage step up method of Benjamini, Krieger, and Yekutieli to correct for multiple comparisons (*p* < 0.05, *q* < 0.1). Differences in muscles weight, fiber type proportion, fiber size distribution, fiber size per type and contents of proteins regulating mitochondrial dynamics were analyzed using an ordinary two-way ANOVA followed by post-hoc tests as described above. Differences in the distribution of shape descriptor values used to assess mitochondrial morphology were tested using a Kolmogorov–Smirnov test comparing cumulative distributions. All statistical analyses were performed using the Prism 7 software (GraphPad, San Diego, CA).

## Results

### Effect of Aging and Caloric Restriction on Body and Muscles Weights

**Figure 1:**
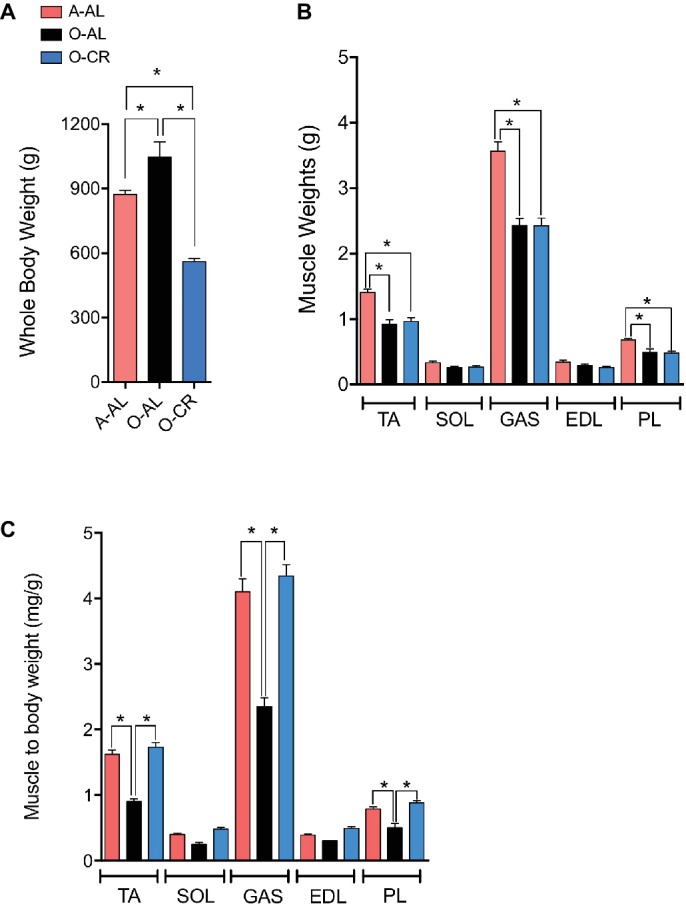
Effect of aging and caloric restriction on body weight and muscles weight. **(A)** Whole body weights and **(B)** TA, SOL, GAS, EDL, PL muscles weights, and **(C)** TA, SOL, GAS, EDL and PL weight relative to body weight of adults (A-AL, N = 9), old ad libitum (O-AL, N = 4) and old CR rats (O-CR, N = 11). Data represented mean ± SEM. *: *p* < 0.05.

O-AL male rats displayed significantly higher body weight vs A-AL rats. As expected, O-CR rats displayed the lowest body weight of our three groups (Figure 1A). No difference in the wet weight of the SOL and EDL was observed among the groups (Figure 1B). The GAS, PL, and TA wet weights were significantly higher in A-AL than in O-CR and O-AL rats (Figure 1B). There was no difference in muscles weights between O-AL and O-CR (Figure 1B). When muscles weights were normalized to body weight, an index of sarcopenia, O-AL rats displayed significantly lower TA, PL, and GAS normalized weights compared to their younger counterparts (Figure 1C). Importantly, O-CR animals displayed significantly higher TA, GAS, and PL normalized weights than O-AL rats. There was no difference in muscle to body weight ratios between O-CR and A-AL rats (Figure 1C), suggesting that CR prevented the aging-related loss of relative muscle mass.

### Effect of Aging and Caloric Restriction on Skeletal Muscle Phenotype

**Figure 2:**
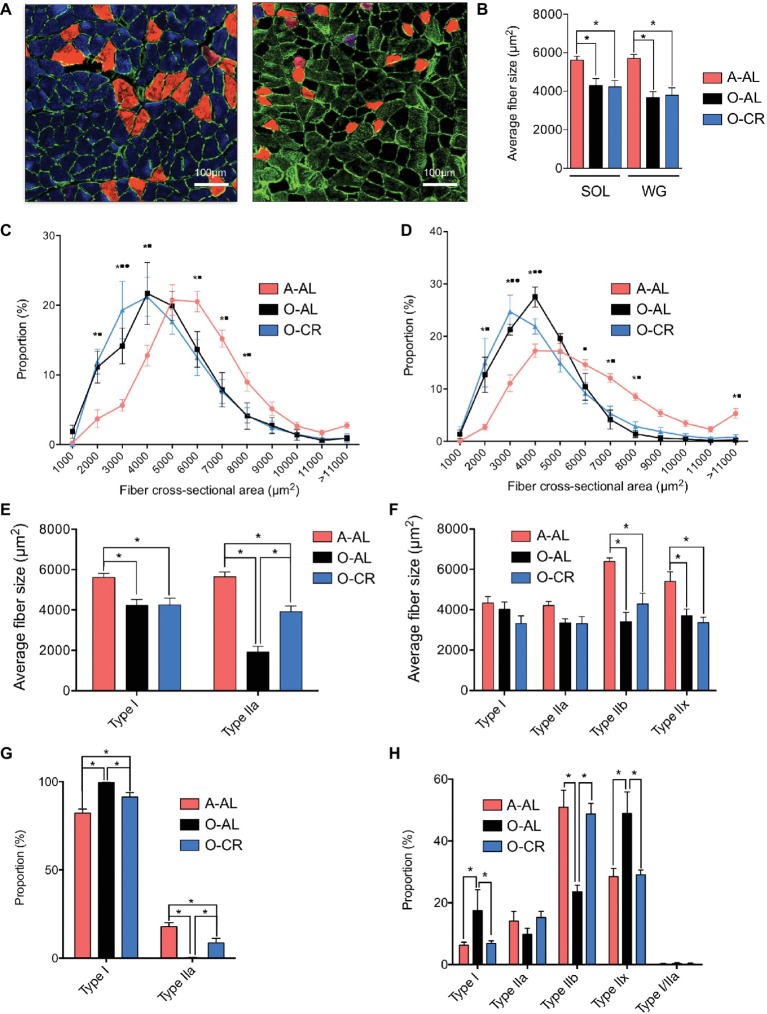
Effect of aging and caloric restriction on fiber cross-sectional area and fibers type. **(A)** Representative immunolabeling of type I (blue), type IIa (red) and type IIb (green) myosin heavy chains (MHCs) performed on muscle cross section of SOL (left) and GAS (right) muscles from A-AL. **(B)** Average fiber size of the SOL and WG of A-AL, O-AL, O-CR rats. **(C)** and **(D)** Fiber size distribution in the SOL **(C)** and GAS **(D)** of A-AL, O-AL and O-CR rats. **(E)** and **(F)** Muscle fiber type composition in SOL **(E)** and GAS **(F)** of A-AL, O-AL and O-CR rats. Muscle fiber type proportion in SOL **(G)** and GAS **(H)** of A-AL, O-AL and O-CR rats. Scale bars: 1000 μm. Data in graphs are presented as Mean ± SEM. *N* = 4 to 9 per group. *: *p* < 0.05. Data in graphs C and D. *: *p* < 0.05 A-AL vs O-AL; ■: *p* < 0.05 A-AL vs O-CR; •: *p* < 0.05 O-AL vs O-CR.

We first investigated the effects of aging and CR on skeletal muscle fiber sizes. To this end, muscle cross sections were immunolabeled for type I, IIa, and IIb MHCs (Figure 2A). Both SOL and GAS muscles in O-AL and O-CR groups displayed significantly lower overall fiber size vs A-AL rats (Figure 2B). A shift to the left of the fiber size distribution in the SOL (Figure 2C) and GAS (Figure 2D) of the O-AL and O-CR rats was observed vs A-AL, indicating an increase in the proportion of small fibers. The SOL muscle in O-AL and O-CR rats displayed significantly smaller type I and IIa fibers compared to that in A-AL rats (Figure 2E). Interestingly, O-CR displayed higher type IIa fiber size vs O-AL, indicating that CR attenuated the effect of aging on type IIa fiber size (Figure 2E). In the case of GAS, no difference in type I and IIa fiber sizes was observed across the tested groups (Figure 2F). However, type IIx and IIb were significantly smaller in O-AL and O-CR vs A-AL (Figure 2F).

The proportions of type I and type IIa fibers in the SOL were respectively higher and lower in O-AL and O-CR vs A-AL (Figure 2G). Interestingly, the proportions of type I and IIa fibers were respectively lower and higher in the SOL of O-CR vs O-AL (Figure 2G). O-AL group displayed significantly higher type I and IIx fiber proportion in the GAS and lower type IIb proportion vs A-AL (Figure 2H). Importantly, O-CR displayed significantly lower type I and IIx fiber proportion and higher type IIb fiber proportion vs O-AL (Figure 2H). Furthermore, no differences in fiber type proportions were observed between O-CR and A-AL (Figure 2H).

Altogether, these data indicate that caloric restriction prevents the effects of aging on muscle fiber type composition.

### Effect of Aging on Skeletal Muscle Mitochondrial Content and Succinate Dehydrogenase Activity

**Figure 3:**
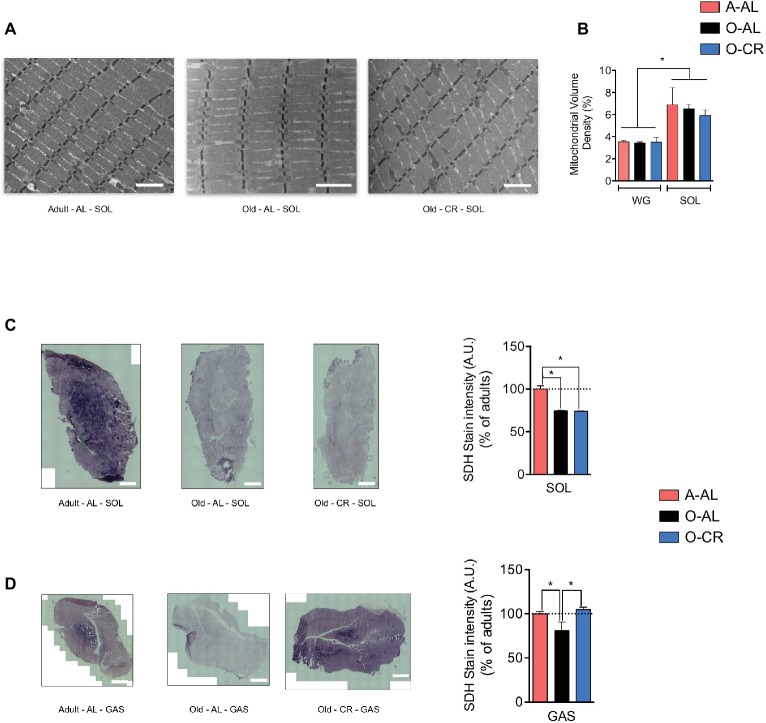
Effect of aging on skeletal muscle mitochondrial content. (A) Representative longitudinal TEM images of A-AL, O-AL and O-CR that were used for the quantification of mitochondrial volume density. The results of these quantifications for the SOL and WG are presented in **(B)** (n = 4 in each group). **(C)** and **(D)** Representative succinate dehydrogenase (SDH) stain and quantification of SOL **(C)** and GAS **(D)** cross-sections of A-AL, O-AL and O-CR (A-AL, N = 7–8; O-AL, N = 4 and O-CR, *N* = 7–8). Data in graphs are presented as Mean ± SEM. Scale bars in **(A)**: 2 μm. Scale bars in **(C)** and **(D)**: 1000 μm.

To assess the effect of aging and CR on skeletal muscle mitochondrial content, we quantified on longitudinal TEM images the mitochondrial density in the SOL and GAS (Figure 3A). As expected, mitochondrial volume density was higher in the SOL vs GAS in all groups (Figure 3B). Interestingly, no difference in mitochondrial volume density was observed between A-AL, O-AL, and O-CR in both muscles (Figure 3B). These results indicate that neither aging nor CR affected mitochondrial content. To assess the impact of aging on mitochondrial enzyme activity, we measured the activity of the Krebs cycle enzyme succinate dehydrogenase (SDH) *in situ* on muscle cross-sections (Figures 3C,D). As shown in Figure 3C, both O-AL and O-CR displayed lower SDH activity in the SOL vs A-AL. Similarly, O-AL displayed lower SDH activity in the GAS vs A-AL (Figure 3D). However, the SDH activity measured in the GAS of O-CR rats was significantly higher than in O-AL but was similar to that of A-AL (Figure 3D).

### Effect of Aging and Caloric Restriction on Skeletal Muscle Mitochondrial Morphology

To define the impact of aging and CR on mitochondrial morphology, shape descriptors were determined from TEM images acquired in both longitudinal and transversal orientations for the 2 populations of mitochondria found in skeletal muscle: subsarcolemmal (SS) and intermyofibrillar (IMF) mitochondria. To specifically assess the effects of aging on mitochondrial morphological complexity, we also plotted the aspect ratio and form factor values for all individual SS and IMF mitochondria. We then determined in A-AL and O-AL and O-CR, the proportion of mitochondria thatwas below the 25^th^ percentile of adult values for both aspect ratio and form factor (morphologically simple). Similarly, we determined in A-AL, O-AL, and O-CR, the proportion of mitochondria that were above the 75^th^ percentile of adult values for both aspect ratio and form factor (morphologically complex).

#### Effects of Aging and CR on Mitochondrial Morphology in the Oxidative SOL Muscle

**Figure 4:**
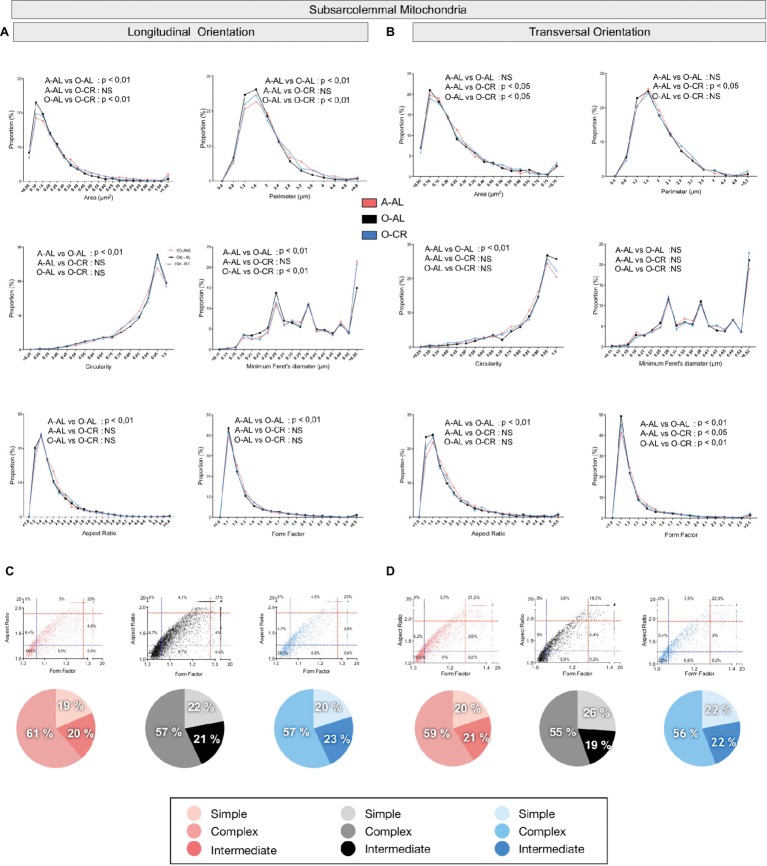
Effect of aging and caloric restriction on the morphology of subsarcolemmal (SS) mitochondria in the oxidative SOL muscle. Analyses of morphological parameters of mitochondria SS in soleus muscle (longitudinal and transversal orientation). **(A)** and **(B)** Frequency distribution of shape descriptors and morphological for SS mitochondria in longitudinal **(A)** and transversal orientations **(B)**. **(C)** and **(D)** display the form factor and aspect ratio relationship for individual SS mitochondria for A-AL (left graph), O-AL (middle graph) and O-CR (right graph) rats in longitudinal (C) and transversal orientations (D). In **(C)** and **(D),** blue and red dashed lines represent the 25th and 75th percentiles for either aspect ratio or form factor values of A-AL, respectively. The pie charts in the second line represent the percentage of mitochondria with simple (i.e. mitochondria with aspect ratio and form factors values inferior to the 25th percentile of A-AL values), complex (i.e. mitochondria with aspect ratio and form factors values above the 75th percentile of A-AL values) and intermediate (i.e. neither simple nor complex). Differences in frequency distributions were tested using a Kolmogorov–Smirnov test comparing cumulative distributions.

When analyzed in longitudinal orientation, SOL muscles from O-AL contained SS mitochondria with significantly lower area, perimeter, and minimum Feret’s diameter than their younger counterparts (Table 1 and Figure 4A). The proportion of SS mitochondria with small aspect ratio and form factor values was also increased in O-AL vs A-AL (Figure 4A). SOL from O-CR displayed SS mitochondria with significantly higher area, perimeter, and minimum Feret’s diameter vs O-AL (Table 1 and Figure 4A). Interestingly, no differences in the distribution of mitochondrial shape descriptors were observed between SS mitochondria from O-CR and A-AL in the SOL in longitudinal orientation (Figure 4A).

In transversal orientation, no significant differences in area, perimeter, and minimum Feret diameter were observed between SS mitochondria from O-AL and A-AL SOL (Figure 4B). However, SS mitochondria from O-AL SOL displayed significantly higher average circularity value (Table 1), a shift to right of the circularity distribution and a shift to the left of the distribution of form factor and aspect ratio values vs A-AL (Figure 4B). As compared to O-AL, SOL from O-CR displayed SS mitochondria with lower average circularity values, higher average form factor (Table 1), as well as a shift to the left of the circularity distribution and a shift to the right of the distribution of form factor values (Figure 4B).

In both transversal and longitudinal orientations, O-AL displayed an increase in the proportion of morphologically simple mitochondria vs A-AL, a difference attenuated in O-CR animals (Figures 4C,D). Taken altogether, the data indicate that aging is associated with a fragmentation of SS mitochondria in the SOL. They also indicate that CR attenuates the effects of aging on SS morphology in the SOL.

**Figure 5:**
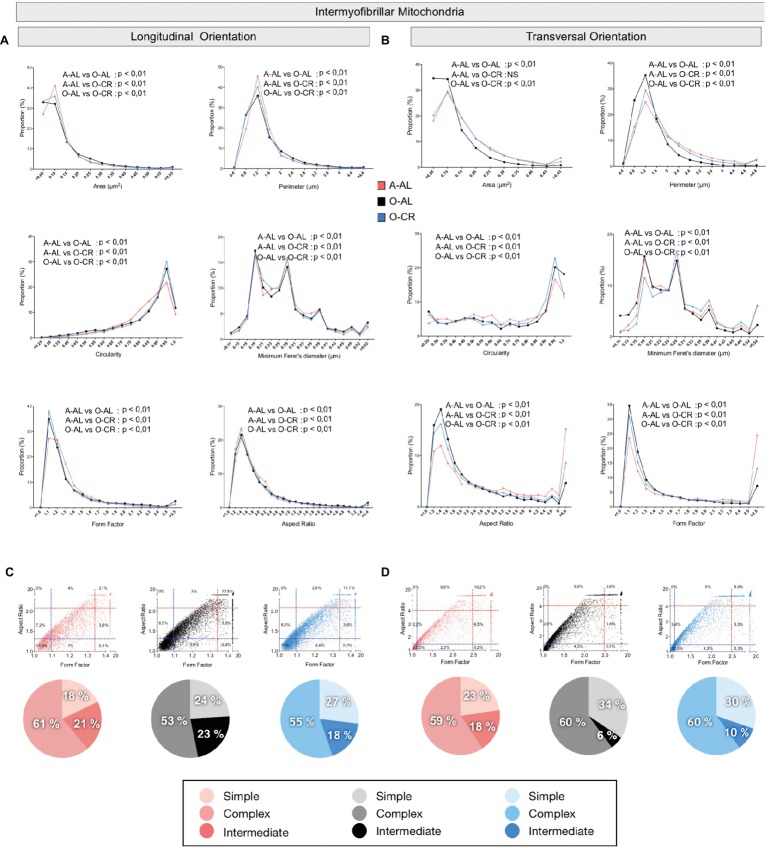
Effect of aging and caloric restriction on the morphology of intermyofibrillar (IMF) mitochondria in the oxidative SOL muscle. Analyses of morphological parameters of mitochondria IMF in the SOL muscle (longitudinal and transversal orientation). **(A)** and **(B)** Frequency distribution of shape descriptors and morphological for IMF mitochondria in longitudinal **(A)** and transversal orientations **(B)**. **(C)** and **(D)** display the form factor and aspect ratio relationship for individual IMF mitochondria for A-AL (left graph), O-AL (middle graph), and O-CR (right graph) rats in longitudinal **(C)** and transversal orientations **(D)**. In **(C)** and **(D),** blue and red dashed lines represent the 25th and 75th percentiles for either aspect ratio or form factor values of A-AL, respectively. The pie charts in the second line represent the percentage of mitochondria with simple (i.e. mitochondria with aspect ratio and form factors values inferior to the 25th percentile of A-AL values), complex (i.e. mitochondria with aspect ratio and form factors values above the 75th percentile of A-AL values) and intermediate (i.e. neither simple nor complex). Differences in frequency distributions were tested using a Kolmogorov–Smirnov test comparing cumulative distributions.

As shown in Figure 5 and Table 1, the average values and frequency distribution of all of the shape descriptors quantified indicate that SOL IMF mitochondria from O-AL rats are fragmented compared to A-AL animals. These data also indicate that CR attenuated aging-related fragmentation of IMF mitochondria in the SOL.

#### Effects of Aging and CR on Mitochondrial Morphology in the Glycolytic WG Muscle

**Figure 6:**
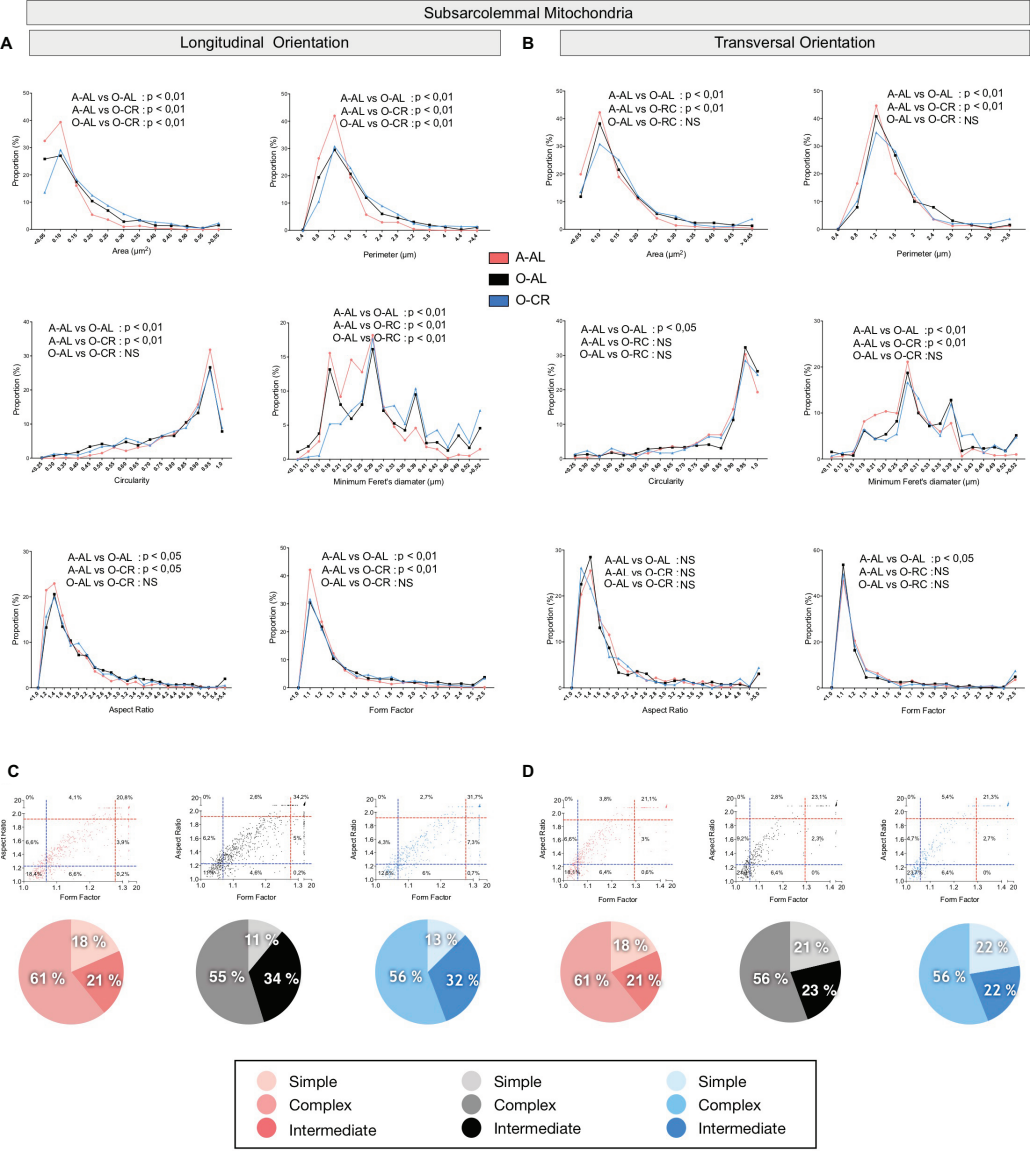
Effect of aging and caloric restriction on the morphology of subsarcolemmal (SS) mitochondria in the glycolytic white gastrocnemius (WG) muscle. Analyses of morphological parameters of mitochondria SS in WG muscle (longitudinal and transversal orientation). **(A)** and **(B)** Frequency distribution of shape descriptors and morphological for SS mitochondria in longitudinal **(A)** and transversal orientations **(B)**. **(C)** and **(D)** display the form factor and aspect ratio relationship for individual SS mitochondria for A-AL (left graph), O-AL (middle graph) and O-CR (right graph) rats in longitudinal **(C)** and transversal orientations **(D)**. In **(C)** and **(D)**, blue and red dashed lines represent the 25th and 75th percentiles for either aspect ratio or form factor values of A-AL, respectively. The pie charts in the second line represent the percentage of mitochondria with simple (i.e. mitochondria with aspect ratio and form factors values inferior to the 25th percentile of A-AL values), complex (i.e. mitochondria with aspect ratio and form factors values above the 75th percentile of A-AL values) and intermediate (i.e. neither simple nor complex). Differences in frequency distributions were tested using a Kolmogorov–Smirnov test comparing cumulative distributions.

In both longitudinal and transversal orientations, SS mitochondria from O-AL animals displayed significantly higher area, perimeter, and minimum Feret’s diameter vs A-AL (Figures 6A,B and Table 2). In addition, SS mitochondria from O-AL animals displayed significantly higher aspect ratio and form factor values, as well as a shift to the right of the circularity distribution in longitudinal orientation vs A-AL (Figure 6A and Table 2). These data associated, with the respective increase and decrease in the proportion of complex and simple SS mitochondria in the WG of O-AL vs A-AL (Figures 6C,D), indicate that old animals display enlarged and more complex SS mitochondria in the WG. As can be seen in Figure 6, as well as in Table 2, CR had marginal anti-aging effects on SS mitochondrial morphology in the WG.

**Table 2:** Effects of aging and caloric restriction on morphological parameters and shape descriptors of subsarcolemmal and intermyofibrillar mitochondria in the white gastrocnemius.

White gastrocnemius						
	Longitudinal orientation			Transverse orientation		
	A-AL	O-AL	O-RC	A-AL	O-AL	O-RC
**SS**
N	610	1,010	559	502	390	117
Area (um^2^)	0.08 ± 0.003^a^^,^^b^	0.14 ± 0.005^a^^,^^c^	0.16 ± 0.006^b^^,^^c^	0.10 ± 0.003^a^^,^^b^	0.13 ± 0.005^a^	0.13 ± 0.010^b^
Perimeter (μm)	1.12 ± 0.019^a^^,^^b^	1.43 ± 0.026^a^^,^^c^	1.59 ± 0.037^b^^,^^c^	1.25 ± 0.026^a^	1.39 ± 0.034^a^	1.34 ± 0.054
Circularity	0.84 ± 0.005^a^^,^^b^	0.77 ± 0.006^a^	0.78 ± 0.007^b^	0.83 ± 0.007	0.83 ± 0.009	0.84 ± 0.015
Aspect ratio	1.69 ± 0.027^a^^,^^b^	2.02 ± 0.034^a^^,^^c^	1.89 ± 0.038^b^^,^^c^	1.82 ± 0.047	1.88 ± 0.070	1.73 ± 0.090
Form factor	1.23 ± 0.011^a^^,^^b^	1.39 ± 0.015^a^	1.37 ± 0.020^b^	1.28 ± 0.019	1.31 ± 0.028	1.28 ± 0.048
Minimum Feret (μm)	0.26 ± 0.003^a^^,^^b^	0.29 ± 0.004^a^^,^^c^	0.33 ± 0.005^b^^,^^c^	0.27 ± 0.003^a^^,^^b^	0.31 ± 0.006^a^	0.32 ± 0.007^b^
**IMF**
N	2,326	3,724	1,566	972	1,352	2,119
Area (um^2^)	0.07 ± 0.001^a^^,^^b^	0.10 ± 0.002^a^^,^^c^	0.12 ± 0.003^b^^,^^c^	0.14 ± 0.004^a^^,^^b^	0.11 ± 0.003^a^^,^^c^	0.10 ± 0.002^b^^,^^c^
Perimeter (μm)	1.00 ± 0.010^a^^,^^b^	1.21 ± 0.011^a^^,^^c^	1.30 ± 0.020^b^^,^^c^	1.73 ± 0.035^a^^,^^b^	1.48 ± 0.027^a^^,^^c^	1.31 ± 0.019^b^^,^^c^
Circularity	0.86 ± 0.003^a^^,^^b^	0.79 ± 0.003^a^^,^^c^	0.82 ± 0.004^b^^,^^c^	0.68 ± 0.008	0.69 ± 0.007	0.70 ± 0.005
Aspect ratio	1.63 ± 0.017^a^^,^^b^	1.97 ± 0.017^a^^,^^c^	1.81 ± 0.026^b^^,^^c^	2.68 ± 0.054^a^	2.96 ± 0.07^a^	2.83 ± 0.049
Form factor	1.21 ± 0.006^a^^,^^b^	1.36 ± 0.007^a^^,^^c^	1.31 ± 0.011^b^^,^^c^	1.78 ± 0.029	1.86 ± 0.035	1.81 ± 0.025
Minimum Feret (μm)	0.23 ± 0.001^a^^,^^b^	0.25 ± 0.001^a^^,^^c^	0.28 ± 0.003^b^^,^^c^	0.30 ± 0.005^a^^,^^b^	0.27 ± 0.004^a^^,^^c^	0.24 ± 0.003^b^^,^^c^

Data are presented as mean ± SEM. Data were obtained from adult ad libitum-fed (A-AL, N = 4), old ad-libitum fed (O-AL, N = 4) and old CR rats (O-CR, N = 4).

a *p* < 0.05 A-AL vs O-AL;

b *p* < 0.05 A-AL vs O-CR;

c *p* < 0.05 O-AL vs O-CR.

**Figure 7:**
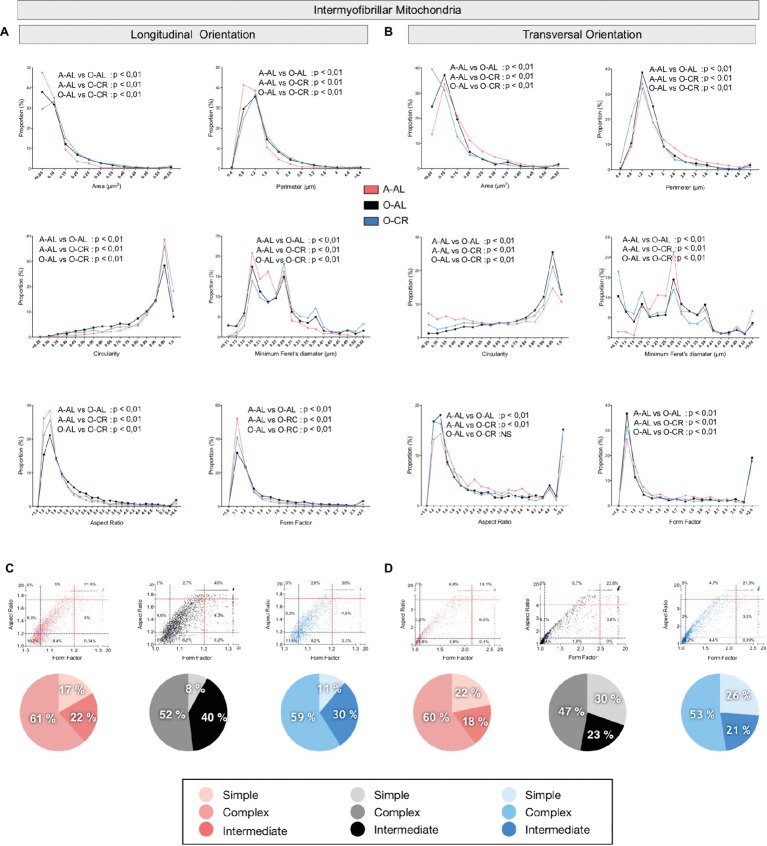
Effect of aging and caloric restriction on the morphology of intermyofibrillar (IMF) mitochondria in the glycolytic white gastrocnemius (WG) muscle. Analyses of morphological parameters of mitochondria IMF in WG muscle (longitudinal and transversal orientations). (**A)** and **(B)** Frequency distribution of shape descriptors and morphological for IMF mitochondria in longitudinal **(A)** and transversal orientations **(B)**. **(C)** and **(D)** display the form factor and aspect ratio relationship for individual IMF mitochondria for A-AL (left graph), O-AL (middle graph) and O-CR (right graph) rats in longitudinal **(C)** and transversal orientations **(D)**. In **(C)** and **(D)**, blue and red dashed lines represent the 25th and 75th percentiles for either aspect ratio or form factor values of A-AL, respectively. The pie charts in the second line represent the percentage of mitochondria with simple (i.e. mitochondria with aspect ratio and form factors values inferior to the 25th percentile of A-AL values), complex (i.e. mitochondria with aspect ratio and form factors values above the 75th percentile of A-AL values) and intermediate (i.e. neither simple nor complex). Differences in frequency distributions were tested using a Kolmogorov–Smirnov test comparing cumulative distributions.

When we analyzed in longitudinal orientation, IMF mitochondria from O-AL rats displayed significantly higher area, perimeter, aspect ratio, form factor, and minimum Feret diameter vs A-AL (Figure 7A and Table 2) and lower circularity values vs A-AL (Figure 7A and Table 2). CR did not attenuate the effects of aging on these shape descriptors in longitudinal orientation; CR even exacerbated the differences between O-AL and A-AL (Figure 7A and Table 2). In contrast to the data obtained in longitudinal orientation, IMF mitochondria from O-AL rats displayed significantly lower area, perimeter, and minimum Feret diameter vs A-AL in transversal orientation (Figure 7A and Table 2). However, IMF mitochondria from O-AL rats in transversal orientation displayed higher form factor values and complex shifts in the distribution of aspect ratio and form factor values vs A-AL (Figure 7A and Table 2). In line with the data obtained in longitudinal orientation, data in transversal orientation indicate that CR did not prevent the effects of aging on all shape descriptors and even exacerbated some differences between O-AL and A-AL (Figure 7B and Table 2). As shown in Figures 7C,D, IMF mitochondria from O-AL rats displayed a higher proportion of morphologically complex mitochondria, especially in longitudinal orientation (Figure 7C). Again, CR has marginal anti-aging effects on the morphological complexity of IMF mitochondrial in the WG (Figures 7C,D).

Overall, these results indicate that aged WG muscles display a higher proportion of enlarged and less circular SS mitochondria and a higher proportion of more complex, elongated, and branched mitochondria. They also indicate that CR exerted minor or no anti-aging effects on mitochondrial morphology in the WG.

### Effects of Aging and Caloric Restriction on Immunoreactive Levels of Proteins Regulating Mitochondrial Dynamics

With the aim of getting a better insight into potential mechanisms underlying the changes in mitochondrial morphology observed in O-AL and O-CR rat skeletal muscles, we next quantified the content of key proteins regulating mitochondrial dynamics.

**Figure 8:**
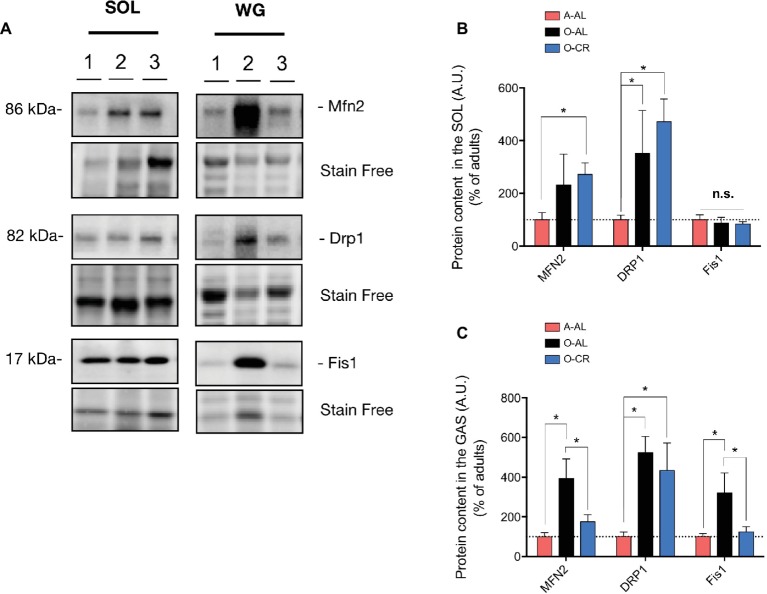
Effect of skeletal muscle aging and caloric restriction on immunoreactive levels of proteins regulating mitochondrial dynamics in oxidative and glycolytic muscles. **(A)** Representative immunoblots of Mfn2, Drp1, Fis1 as well as their corresponding stain free images. **(B,C)** Quantification of Mfn2, Drp1, Fis1 proteins contents (normalized to their corresponding to their stain free intensity) in the SOL (B) and GAS (C) of adult (A-AL), old ad libitum-fed (O-AL) and old CR (O-CR) rats. Data in graphs are presented as Mean ± SEM (*n* = 7–8 A-AL, *n* = 4 O-AL, *n* = 8–10 O-CR per group. *p < 0.05).

As shown in Figures 8A,B, the immunoreactive levels of the pro-fission protein Drp1 were significantly higher in the SOL of O-AL vs A-AL, while no difference was observed between O-AL and A-AL for the contents of Mfn2 and Fis1. Surprisingly, O-CR displayed higher Drp1 content vs A-AL (Figures 8A,B). While no difference was observed between O-CR and A-AL for Fis1 content, the Mfn2 level was significantly higher in the SOL of O-CR vs A-AL (Figures 8A,B).

In the GAS, O-AL displayed significantly higher Mfn2, Drp1, and Fis1 protein levels vs A-AL (Figures 8A,C). Protein contents of Mfn2 and Fis1 were also higher in the GAS of O-AL vs O-CR (Figures 8A,C). The WG of O-CR displayed higher contents of Drp1 vs A-AL rats (Figures 8A,C). No difference in Mfn2 and Fis1 contents was observed in the GAS of O-CR vs A-AL rats (Figures 8A,C).

Taken altogether, our data indicate that aging increases mitochondrial fission in the SOL while increasing both mitochondrial fission and fusion in the GAS. They also indicate that CR partially attenuated the impact of aging on mitochondrial dynamics in the GAS and might have exerted a pro-fusion impact in the SOL.

## Discussion

The aim of the present study was to define the impact of aging and caloric restriction on mitochondrial morphology and dynamics in oxidative and glycolytic muscles. Here, we first show that CR attenuated the impact of aging on muscle mass to body weight ratio, a sarcopenic index. We also provide evidence that CR abolished the effects of aging on muscle fiber type composition in both types of muscle. To assess the impact of aging and calorie restriction on mitochondrial morphology, we used a quantitative 2-dimensional electron microscopy approach to evaluate the morphology of the two populations of skeletal muscle mitochondria, IMF and SS, in the SOL and WG muscles. Using this approach, and in contrast with our initial hypotheses, our results indicate that aging has opposite effects on mitochondrial morphology in the SOL vs WG muscles. Indeed, we show that aging in the SOL is associated with a fragmentation of SS and IMF mitochondria. In contrast, we found that aging in the highly glycolytic WG is associated with an enlargement of SS mitochondria and an increase in the complexity and branching of IMF mitochondria. We also show that CR attenuated this aging-related fragmentation of SS and IMF mitochondria in the SOL but had little to no impact on mitochondrial morphology in the WG. We finally show that while aging is associated with an increase in DRP1 (pro-fission) in the SOL and with increases in Mfn2 (pro-fusion), Drp1, and Fis1 (pro-fission) in the GAS, CR resulted in elevated levels of Mfn2 in the SOL and attenuated the aging-related increase in Mfn2 and Fis1 contents in the GAS.

### Effect of Aging and Caloric Restriction on Skeletal Muscle Mass and Phenotype

Although CR did not attenuate the effect of aging on skeletal muscle mass, it fully prevented the impact of aging on the muscle weight to body weight ratio, a widely used index of sarcopenia (Edstrom et al., 2006; Paturi et al., 2010; Ezzat-Zadeh et al., 2017). The preservation of this index suggests that CR attenuated the impact of aging on animal mobility and functional capacities, a speculation consistent with previous literature showing a protective impact of CR on muscle function (Hepple et al., 2005) and functional capacities (Salvatore et al., 2016). Although no effect of CR was observed in muscle fiber size, CR also totally prevented the impact of aging on muscle fiber type composition. Indeed, O-AL rats displayed a decrease in type IIa fiber proportion in the SOL and a shift toward more oxidative fibers in the GAS. In contrast, no difference in muscle fiber type composition was observed between O-CR and A-AL. The current theory explaining the progressive remodeling of muscle fiber type composition occurring in aging is based on the fact that skeletal muscle fibers undergo progressive cycles of denervation and reinnervation with age. This theory, supported by ample scientific evidence (see (Gouspillou et al., 2013) for a detailed review), implicates that some fibers will lose their innervation, due to impaired neuromuscular integrity, before being re-innervated by axonal sprouting from an adjacent motor neuron. This process is believed to progressively remodel muscle fiber type composition with age, and atrophy is thought to occur when the rate of denervation outpaces the rate of reinnervation. The absence of difference in muscle fiber type composition in O-CR vs A-AL SOL suggests that CR might protect the integrity of neuromuscular junctions (NMJ) during the aging process. This is in line with the results from Valdez et al., showing that CR significantly reduced the incidence of NMJ abnormalities in 24-month-old mice and attenuated aging-related loss of motor neurons (Valdez et al., 2010). Although muscle contractility was not assessed in the present study, one might speculate that the protective impact of CR against aging-related changes in muscle fiber type composition might have translated into a protective impact on muscle contractility. Such a speculation is in agreement with a previous report showing that CR attenuates the aging-related decline in muscle specific strength (Hepple et al., 2006).

### Effect of Aging and Caloric Restriction on Mitochondrial Content

The impact of aging on muscle mitochondrial content remains a controversial topic, with some studies showing reduced mitochondrial content in aged skeletal muscle (Conley et al., 2000; Chabi et al., 2008; Lanza and Nair, 2009; Crane et al., 2010) and others reporting no effect of aging (Russ and Kent-Braun, 2004; Gouspillou et al., 2014b; Leduc-Gaudet et al., 2015; St-Jean-Pelletier et al., 2017). The present study supports the view that aging is not associated with a decline in mitochondrial content, since the mitochondrial density, assessed here using the gold standard approach (i.e. assessed on TEM images), was not reduced in both the SOL and O-AL rats. However, we found that O-AL animals display a decrease in SDH activity in both the SOL and GAS muscles, suggesting that although mitochondrial content was preserved in muscle of O-AL rats, mitochondrial bioenergetics might be impaired in old muscles. Although CR has been proposed to increase mitochondrial content (Guarente, 2008), no impact of CR on mitochondrial density could be evidenced in the present study. The absence of effect of CR on mitochondrial content in aged skeletal muscle is in line with recent studies from the Hollozy and Nair research groups, which have shown that CR does not increase mitochondrial content in rodent skeletal muscle (Hancock et al., 2011; Lanza et al., 2012). Interestingly, Lanza et al. have also shown that CR attenuates the effects of aging on state 3 (ADP-stimulated) mitochondrial respiration in the GAS (Lanza et al., 2012). This result is in line with our present finding showing that CR attenuates the effect of aging on the SDH activity in the GAS. The present results show that CR did not protect against the effects of aging on SDH activity in the SOL, suggesting that not all muscles are protected from the aging-induced decline in mitochondrial function by CR. The mechanism underlying the differential effect of CR on SDH activity in the SOL and GAS requires further study.

### Effect of Aging and Caloric Restriction on Mitochondrial Morphology and Dynamics

Few studies have investigated the effects of aging on mitochondrial morphology in skeletal muscle but their results were contradictory. While some studies have provided data suggesting that aging is associated with a fragmentation of IMF mitochondria (Poggi et al., 1987; Huang et al., 2010; Lanza et al., 2012; Iqbal et al., 2013), others have provided data indicating that aging is associated with an increased size of IMF mitochondria (Sebastian et al., 2016), an increased branching and morphological complexity of IMF mitochondria (Leduc-Gaudet et al., 2015), an enlargement of SS mitochondria (Leduc-Gaudet et al., 2015), and an increase in mitochondrial fusion (Leduc-Gaudet et al., 2015; Picca et al., 2017). Importantly, all studies reporting an aging-related decrease in IMF mitochondrial size only assessed mitochondrial morphology in longitudinal orientation. Sectioning skeletal muscle in the transverse orientation is however essential to accurately quantify IMF mitochondrial morphology (Ogata and Yamasaki, 1997; Picard et al., 2013b). This is because IMF mitochondria are tubular and branched structures that align along the sarcomeric plan (in transverse orientation) (Ogata and Yamasaki, 1997), which essentially appear as round structures when visualized in longitudinal orientation (Gouspillou et al., 2013). In addition, to our knowledge, no previous study has compared the impact of aging and CR, on both oxidative and glycolytic muscles. The latter point is particularly important since mitochondrial function is well known to differ between oxidative and glycolytic muscles (Picard et al., 2012). It is therefore possible that mitochondrial morphology, and the impact of aging and CR, might also differ between oxidative and glycolytic muscles. Taking these factors into consideration, the present study is the most thorough investigation of mitochondrial morphology in the context of aging, since multiple representative shape descriptors were quantified in longitudinal and transverse orientations for both SS and IMF mitochondria and in both glycolytic and oxidative muscles. It is therefore possible that the opposite effects we report here of aging on mitochondrial morphology (i.e., fragmentation of SS and IMF mitochondria in the SOL and enlargement of SS mitochondria and an increase in the complexity and branching of IMF mitochondria in the WG) might explain some of the discrepancy currently present in the literature. In addition, our results highlight that while CR seems to protect against the effect of aging on mitochondrial morphology in the SOL, it had minor or no anti-aging effect on mitochondrial morphology in the glycolytic WG.

Our data on mitochondrial dynamics indicate that aging in the SOL is associated with an increase in the content of the pro-fission protein Drp1. A result consistent with the increase in mitochondrial fragmentation we observed in this oxidative muscle. Although CR did not attenuate the effects of aging on Drp1 content in the SOL, it was associated with an increase in the content of pro-fusion protein Mfn2. This increase in Mfn2 content seen in the SOL of O-CR animals might be involved in the protective effects of CR against the aging-related increase in mitochondrial fragmentation in the SOL. The enlargement of SS mitochondria and the increase in the complexity and branching of IMF mitochondria seen in the WG of O-AL rats were associated with an increase in Mfn2, as well as an increase in the pro-fission protein Drp1 and Fis1. While the higher content of Mfn2 is in line with our morphological data and previous studies that have suggested an increase in mitochondrial fusion in aged muscle (Leduc-Gaudet et al., 2015; Picca et al., 2017), the concomitant increase in Drp1 and Fis1 also suggests enhanced mitochondrial fission in the WG of aged rats. This concomitant increase in the expression of both pro-fusion and pro-fission proteins might reflect an attempt to compensate for an aging-related increase in mitochondrial dysfunction by (1) increasing fusion to mix the matrix content of healthy mitochondria with that of dysfunctional mitochondria in order to dampen mitochondrial dysfunction (Chen et al., 2010) and (2) increase fission to segregate the most dysfunctional mitochondria to ultimately degrade them *via* autophagy (Twig et al., 2008). Whether these speculations are true or whether these changes are causally involved in the accumulation of mitochondrial dysfunction requires further mechanistic study to investigate the impact of modulating both mitochondrial fusion and fission processes on skeletal muscle aging. Interestingly, while CR did not attenuate the effect of aging on mitochondrial morphology, it did attenuate the impact of aging on the expression of some proteins regulating mitochondrial dynamics. These findings, associated with our data showing that CR attenuated the aging-related decline in SDH activity in the GAS, suggest that the CR-induced decrease in Mfn2 and Fis1 content might have played a protective role against the aging-related decrease in SDH activity.

## Conclusions

The present study provides evidence that aging-related muscle atrophy is associated with differential changes in mitochondrial morphology and dynamics in oxidative vs glycolytic muscle fibers. Indeed, we show that oxidative fibers display increased mitochondrial fission/fragmentation, and glycolytic fibers display increased mitochondrial size and branching. Our results also indicate that although CR partially attenuates aging-related changes in the content of proteins regulating mitochondrial dynamics in glycolytic fibers, its anti-aging effect on mitochondrial morphology is restricted to oxidative fibers. These results therefore highlight the critical need to analyze both glycolytic and oxidative muscles to reach complete and accurate conclusions on the effects any intervention, condition, genetic manipulation, or treatment on skeletal muscle mitochondria.

## Ethics Statement

All animal procedures were approved by the *Comité Institutionnel de Protection des Animaux de l’UQAM* (#CIPA875) and the *Centre de recherche de l’hôpital du Sacré-Coeur de Montréal* (#FRSQ.01) in compliance with the guidelines of the Canadian Council on Animal Care.

## Author Contributions

JF and GG contributed to the conception and design of the study. JF, J-PL-G, OR and GG collected the data. All authors contributed to data analysis and interpretation. JF and GG wrote the first draft of the manuscript. All authors contributed to manuscript revision, read, and approved the submitted version.

### Conflict of Interest Statement

The authors declare that the research was conducted in the absence of any commercial or financial relationships that could be construed as a potential conflict of interest.
